# Pharmacological Trends in the Management of Atopic Dermatitis: A Comprehensive Review

**DOI:** 10.7759/cureus.64302

**Published:** 2024-07-11

**Authors:** Drishti M Bhatt, Adarshlata Singh, Bhushan Madke, Shivani D Jangid, Talasila Sree Ramya

**Affiliations:** 1 Dermatology, Jawaharlal Nehru Medical College, Datta Meghe Institute of Higher Education and Research, Wardha, IND

**Keywords:** treatment efficacy, personalized medicine, interleukin-4 (il-4) and il-13 inhibitors, janus kinase inhibitors (jak inhibitors), biologic therapies, atopic dermatitis

## Abstract

Atopic dermatitis (AD) is a prevalent, chronic inflammatory skin condition characterized by pruritus, erythema, and impaired skin barrier function. AD management presents significant challenges due to its complex pathophysiology involving immune dysregulation and genetic predispositions. While traditional therapies, such as topical corticosteroids and emollients, remain foundational, their limitations have spurred the development of novel pharmacological approaches. This comprehensive review explores current pharmacological trends in the management of AD, focusing on emerging therapies that target specific immunological pathways. Biologic agents, including monoclonal antibodies against interleukin (IL)-4, IL-13, and IL-31 receptors, offer targeted mechanisms to modulate immune responses implicated in AD pathogenesis. Janus kinase (JAK) and phosphodiesterase-4 (PDE-4) inhibitors represent another class of promising therapies, providing alternatives for patients resistant to conventional treatments. The review synthesizes evidence from clinical trials and studies to evaluate these pharmacological agents' efficacy and safety profiles. Considerations for personalized medicine approaches, including biomarkers for treatment response prediction and genotype-based therapies, are discussed to highlight the potential for tailored treatment strategies in AD management. In conclusion, this review underscores the evolving landscape of pharmacological interventions for AD, emphasizing the need for continued research to address unmet clinical needs and optimize patient outcomes. By delineating current advancements and future directions, this review aims to inform clinical practice and guide future research endeavours in dermatology.

## Introduction and background

Atopic dermatitis (AD), a chronic inflammatory skin condition characterized by pruritus, erythema, and lichenification, poses a significant burden on patients' quality of life and healthcare systems worldwide [1]. As one of the most prevalent dermatological disorders, AD affects individuals of all ages, with a notable rise in paediatric cases in recent years. The multifaceted nature of AD, involving complex immunological pathways and genetic predispositions, underscores the need for effective pharmacological management strategies [2].

Effective management of AD often requires a multifaceted approach, integrating lifestyle modifications, topical therapies, and, in more severe cases, systemic pharmacotherapy. This review aims to comprehensively explore the current pharmacological landscape for AD, emphasizing emerging trends and novel therapeutic approaches [3]. By synthesizing existing knowledge and highlighting recent advancements, this review seeks to provide a critical analysis of pharmacological interventions aimed at improving outcomes for AD patients [4].

The scope of this review encompasses an in-depth examination of biological therapies, Janus kinase (JAK) inhibitors, phosphodiesterase-4 (PDE-4) inhibitors, and other promising pharmacological agents. Additionally, the review will discuss the clinical efficacy, safety profiles, and potential for personalized medicine in treating AD. This review aims to contribute to the evolving understanding and management of AD by addressing these aspects, offering insights into future research directions and clinical practices.

## Review

Current treatment landscape

Traditional Therapies (Topical Corticosteroids (TCS) and Emollients)

TCS are the first-line treatment for mild-to-moderate AD. They effectively reduce inflammation and itching, but long-term use can lead to side effects such as skin thinning [5,6]. Consequently, physicians often prescribe topical steroids only for acute flare-ups to minimize these risks [7]. Emollients and moisturizers are essential components in managing AD. They help restore the skin’s barrier function, prevent flare-ups, and reduce the need for topical steroids [6,8]. Emollients include creams, ointments, and oils like sunflower seed and virgin coconut oil, which possess anti-inflammatory and skin-healing properties [8]. Topical calcineurin inhibitors (TCIs), such as tacrolimus and pimecrolimus, represent another class of topical medications used for AD. They effectively reduce inflammation by inhibiting T and mast cells [7]. However, their long-term safety is still under evaluation, and they carry an FDA black box warning for potential cancer risk [5,6]. For severe, treatment-resistant cases, systemic immunosuppressant medications may be employed. These include oral steroids, cyclosporine, methotrexate, azathioprine, and mycophenolate mofetil [5,7,9]. While these drugs are effective, they have significant side effects and are not suitable for long-term use [7]. Traditional therapies, such as topical steroids, emollients, and calcineurin inhibitors, remain the cornerstone of AD treatment, with systemic immunosuppressants reserved for the most severe cases. However, there is an ongoing need for safer and more effective long-term treatment options.

Limitations and Challenges of Current Treatments

TCS, while effective, are often met with patient hesitation due to concerns about steroid phobia, skin thinning, and rebound effects [10,11]. TCIs, like tacrolimus and pimecrolimus, carry a black box warning due to potential cancer risks, which limits their use to second-line therapy [10,11]. Systemic immunomodulators, such as cyclosporine, methotrexate, and azathioprine, have been employed for severe refractory disease, but they come with significant side effects and are not FDA-approved for AD [5]. Newer biologic therapies, like dupilumab, are highly effective but require injections, which can be a barrier for some patients [10,11]. Many patients struggle with the time-consuming nature of applying topical medications to large body surface areas, especially paediatric patients who require parental assistance [11]. There is also a notable gap in treatment options for patients with severe disease requiring systemic therapy and those with milder disease requiring topical therapy [10]. Cycling within and between both topical and systemic drug classes is common, particularly in patients with more severe disease, indicating the difficulty in managing these cases and highlighting the need for more effective treatment options [12]. The current treatment landscape is expanding with the introduction of targeted biologics and emerging JAK inhibitors. Still, challenges remain in providing safe, effective, and convenient therapies for all patients across the spectrum of disease severity.

Need for Alternative Pharmacological Approaches

Current therapies for AD, such as TCS and calcineurin inhibitors, exhibit notable limitations despite their effectiveness. Issues like patient reluctance due to steroid phobia, potential side effects such as skin thinning and cancer risks associated with calcineurin inhibitors, and variable treatment efficacy underscore the urgent need for therapies that are not only highly effective but also fast-acting and possess a favourable safety profile [11,12]. As the prevalence of AD continues to rise, a significant portion of patients are experiencing moderate-to-severe forms of the disease that are challenging to manage with conventional treatments [13]. This increasing severity emphasizes the need for innovative treatment options to address these patients' unmet needs better.

AD is a complex and heterogeneous condition with diverse phenotypic and endotypic variations. This variability complicates treatment outcomes, highlighting the inadequacy of a uniform treatment approach and underscoring the demand for personalized therapies tailored to individual patient profiles [13]. Emerging therapies, such as JAK inhibitors and biologics, represent promising avenues for improved treatment efficacy and safety in AD. Clinical trials have shown encouraging results, suggesting that these new treatments could offer superior outcomes compared to current standards [11]. For instance, topical JAK inhibitors, such as ruxolitinib, are being studied for their ability to combine the rapid effectiveness of corticosteroids with the safety profile of nonsteroidal agents, potentially offering a breakthrough in managing the disease [12].

**Figure 1:**
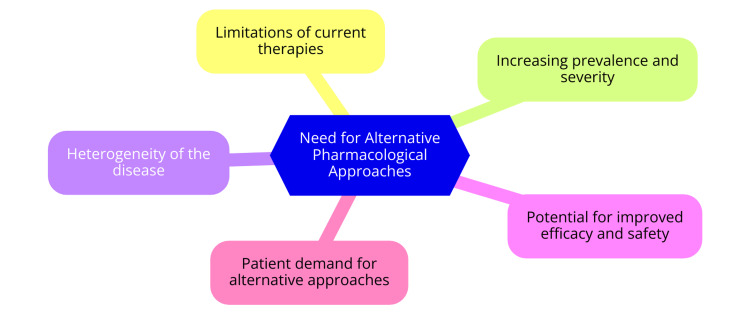
Need for alternative pharmacological approaches Image credit: Dr. Drishti Bhatt

Beyond conventional medical treatments, there is a growing trend among AD patients to seek alternative and complementary therapies, like Chinese herbal medicine, acupuncture, and dietary modifications. This shift reflects dissatisfaction with existing treatment options and a desire for more natural or holistic approaches to managing their condition [14]. While the scientific evidence supporting these alternative therapies remains limited, their popularity underscores the importance of expanding the range of treatment choices available to patients, ensuring that individual preferences and needs are adequately met in managing AD. The need for alternative pharmacological approaches is shown in Figure 1.

Emerging pharmacological trends

Biologic Therapies (Monoclonal Antibodies Targeting IL-4, IL-13, IL-31 Receptors)

In the realm of AD treatment, targeted biological therapies have emerged as pivotal advancements. Dupilumab, an anti-IL-4Rα monoclonal antibody, has demonstrated unprecedented efficacy in clinical trials. Studies indicate that 36-38% of patients achieve clear or nearly clear skin when treated with dupilumab, contrasting significantly with the 8-10% response rate observed in placebo groups [15,16]. Notably, dupilumab's approval for children as young as six months underscores its broad applicability across different age groups, marking a significant step forward in paediatric dermatology. Immunomodulators, like TCIs such as tacrolimus and pimecrolimus, offer another avenue of treatment, particularly for cases resistant to traditional therapies. These agents have shown effectiveness in managing AD symptoms, albeit with caution, due to a black box warning highlighting potential cancer risks [16]. Consequently, they are recommended as second-line options, ensuring their use is carefully weighed against their associated safety concerns.

The emergence of JAK inhibitors, such as baricitinib, represents a promising development in AD therapeutics. These inhibitors target critical inflammatory pathways involved in the disease process, presenting a novel approach to treatment. Clinical trials have demonstrated encouraging results, suggesting that JAK inhibitors could provide effective relief for patients who do not respond adequately to existing therapies [16]. Combination therapies, which integrate TCS with systemic biologics like dupilumab, have shown enhanced efficacy compared to single-agent approaches. This strategy leverages the rapid symptomatic relief offered by corticosteroids alongside the disease-modifying effects of biologics, potentially improving overall treatment outcomes for patients with moderate-to-severe AD [15,16]. This integrated approach reflects a growing understanding of the multifaceted nature of the disease and aims to optimize therapeutic benefits while minimizing adverse effects.

Furthermore, precision medicine is gaining traction in managing AD. Acknowledging the disease's heterogeneous nature, precision medicine endeavours to tailor treatments based on individual patient characteristics, including specific phenotypes and underlying molecular pathways [15,16]. By targeting treatments more precisely to the unique profiles of patients, this approach holds promise for achieving long-term disease control and improving quality of life, marking a significant evolution in how AD is managed clinically.

JAK Inhibitors

JAK inhibitors represent a burgeoning class of targeted therapies showing considerable potential in treating various autoimmune and inflammatory disorders, including AD. These inhibitors block enzymes within the JAK family-JAK1, JAK2, JAK3, and TYK2-thus disrupting the JAK-STAT signalling pathway crucial for regulating immune responses [17]. Several JAK inhibitors are currently under development or have been approved to treat AD, such as baricitinib, upadacitinib, abrocitinib, and delgocitinib [18]. These medications have exhibited efficacy in clinical trials, particularly in managing moderate-to-severe forms of the disease. Ruxolitinib, a JAK1/2 inhibitor, received FDA approval in 2021 for the topical treatment of mild to moderate AD and is also being explored for its effectiveness in treating vitiligo [19]. JAK inhibitors are classified based on their specificity, encompassing pan-JAK inhibitors, selective JAK1 inhibitors, JAK1/2 inhibitors, JAK1/3 inhibitors, JAK3 inhibitors, and TYK2 inhibitors, each tailored to modulate immune responses in distinct ways [19]. Beyond AD, ongoing research is investigating their potential applications in other autoimmune skin conditions like psoriasis, vitiligo, and chronic hand dermatitis [19]. While JAK inhibitors offer a targeted approach to immune modulation, concerns regarding their safety persist. Issues such as heightened susceptibility to infections, potential malignancy risks, and thrombotic events have been noted [19]. Therefore, careful patient selection and vigilant monitoring are essential considerations in their clinical use to mitigate these risks effectively. As research continues to refine our understanding of their benefits and risks, JAK inhibitors hold promise as a significant therapeutic advancement in managing complex dermatologic conditions.

PDE-4 Inhibitors

PDE-4 inhibitors are pharmaceutical agents that exert their therapeutic effects by blocking the degradation of cyclic adenosine monophosphate (cAMP), an essential intracellular signalling molecule, in regulating inflammation. By inhibiting the breakdown of cAMP, these inhibitors effectively suppress inflammatory responses within the body, offering a targeted approach to managing various inflammatory conditions [20]. This mechanism of action has broad implications across diverse disease areas, including central nervous system disorders, asthma, chronic obstructive pulmonary disease (COPD), rheumatoid arthritis, and notably, AD [21]. In the context of AD, PDE-4 inhibitors like crisaborole have garnered attention for their ability to alleviate symptoms by reducing inflammation at the skin level [22]. The therapeutic utility of PDE-4 inhibitors extends beyond their current applications, with ongoing research focusing on their potential in treating inflammatory skin conditions. Emerging agents such as apremilast and roflumilast are either in development or have already been approved for conditions like psoriasis and AD [23]. These new treatments offer promising alternatives by targeting the inflammatory pathways underlying these diseases, potentially providing patients with more effective and better-tolerated treatment options. Combination therapy strategies involving PDE-4 inhibitors and other treatments, such as TCS, have shown enhanced efficacy compared to single-agent approaches in managing inflammatory skin conditions [24]. This synergistic approach capitalizes on the rapid anti-inflammatory effects of corticosteroids and the sustained modulation of inflammatory responses provided by PDE-4 inhibitors. Such combinations improve symptom control and address the multifaceted nature of inflammatory diseases, aiming to optimize patient outcomes through integrated therapeutic strategies. As research advances in this field, the role of PDE-4 inhibitors in treating inflammatory conditions is expected to evolve, potentially reshaping the landscape of dermatologic therapy with safer, more effective treatments.

Novel Small Molecule Inhibitors and Immunomodulators

Targeted biologic therapies have significantly advanced the treatment landscape for AD, with dupilumab, an anti-IL-4Rα monoclonal antibody, leading the forefront. Clinical trials have demonstrated dupilumab's remarkable efficacy, with 36-38% of patients achieving clear or nearly clear skin, starkly contrasting with the 8-10% seen with placebo [25]. The approval extension to include adolescents and children as young as six years old underscores its broad therapeutic impact across different age groups, marking a significant advancement in paediatric dermatology. This expansion highlights dupilumab's effectiveness and pivotal role in providing much-needed relief for younger patients facing the challenges of AD. Immunomodulators like TCIs tacrolimus and pimecrolimus offer additional treatment options particularly suited for moderate-to-severe cases resistant to conventional therapies such as corticosteroids. These inhibitors have shown efficacy in managing symptoms; however, their use is tempered by a black box warning due to potential cancer risks [26]. This precautionary label positions them as second-line therapies, necessitating careful consideration of their benefits weighed against associated safety concerns in clinical practice.

The emergence of JAK inhibitors like baricitinib represents a promising frontier in AD treatment. By targeting key inflammatory pathways, JAK inhibitors offer a novel therapeutic approach that has shown encouraging results in clinical trials [27]. This class of medications holds the potential to address the needs of patients who do not respond adequately to existing treatments, potentially reshaping therapeutic strategies in the management of AD. Combination therapies, such as integrating TCS with systemic biologics like dupilumab, have demonstrated enhanced efficacy compared to monotherapy approaches [28]. This synergistic approach leverages corticosteroids' rapid relief with biologics' disease-modifying effects, aiming to optimize treatment outcomes for patients with moderate-to-severe AD. Such integrated strategies highlight the evolving understanding of the disease's complexity and the ongoing efforts to refine treatment protocols to achieve better patient outcomes. Recognizing AD as a heterogeneous condition, the adoption of precision medicine approaches is gaining momentum. Tailoring treatments based on specific patient phenotypes and molecular profiles holds promise for delivering more effective and personalized care [29]. This shift towards precision medicine aims to improve therapeutic outcomes and minimize adverse effects associated with broad-spectrum treatments, paving the way for more targeted and individualized approaches to managing AD.

Clinical efficacy and safety profiles

Review of Clinical Trials and Studies for Each Class of Pharmacological Agents

In AD treatment, targeted biological therapies have revolutionized therapeutic approaches, with dupilumab emerging as a standout. This anti-IL-4Rα monoclonal antibody has demonstrated unprecedented efficacy in clinical trials, showing that 36-38% of patients achieve clear or nearly clear skin, surpassing the 8-10% response rate seen with placebo [30,31]. Its recent approval extension to include children as young as six years old underscores its efficacy and safety across a wide age range, marking a significant advancement in paediatric dermatology. Alongside dupilumab, tralokinumab, an anti-IL-13 inhibitor, has shown sustained efficacy and acceptable safety over up to four years in adults with moderate-to-severe AD, contributing to the expanding arsenal of targeted biologic therapies [31]. Furthermore, the approval of lebrikizumab in Europe in 2023 further diversifies treatment options, highlighting the evolving landscape of biological treatments tailored to address specific aspects of AD [31]. JAK inhibitors represent another pivotal class of therapies in the AD treatment paradigm. Baricitinib, a JAK1/2 inhibitor, received European approval in 2020 based on its ability to rapidly alleviate skin symptoms and improve overall quality of life for patients [31]. Studies exploring its combination with topical steroids have demonstrated synergistic benefits, enhancing symptom control, quality of life, and functional outcomes in adults with moderate-to-severe disease [31]. Upadacitinib and abrocitinib, both JAK1 inhibitors, have also shown promising short-term efficacy among approved systemic therapies, highlighting their potential role in managing the complex inflammatory pathways involved in AD [31]. Numerous emerging therapies are advancing through phase III clinical trials, aiming to diversify treatment options further and address unmet AD management needs. These include innovative topical agents such as tapinarof, ruxolitinib, delgocitinib, and PDE-4 inhibitors like roflumilast and difamilast, all targeting specific aspects of the disease's pathophysiology [31]. Systemic therapies in late-stage trials encompass novel approaches like cord-blood-derived mesenchymal stem cells, CM310, nemolizumab, anti-OX40/OX40L antibodies, and neurokinin-receptor-1 antagonists, each aiming to modulate immune responses and reduce disease burden in patients with severe or refractory AD [31]. These developments underscore a promising future for personalized and effective treatments, emphasizing a tailored approach to address the heterogeneous nature of AD and improve outcomes for affected individuals.

Comparative Efficacy Against Traditional Treatments

Dupilumab, an anti-IL-4Rα monoclonal antibody, has demonstrated exceptional effectiveness in clinical trials, where 36-38% of patients achieved clear or nearly clear skin compared to only 8-10% with placebo [32]. Its approval has been expanded to encompass adolescents and children as young as six years old, reflecting its broadened applicability across different age groups [32]. In a network meta-analysis, dupilumab could offer comparable efficacy to higher-dose cyclosporine and superior efficacy to methotrexate and azathioprine [32]. Several JAK inhibitors, including baricitinib, abrocitinib, and upadacitinib, are emerging as promising new therapies with enhanced efficacy compared to placebo [32,33]. A real-world study investigating time to remission (Investigator's Global Assessment (IGA) 0/1) reported hazard ratios of 4.2 for oral cyclosporine, 5.05 for dupilumab, and 67.56 for oral JAK inhibitors compared to topical treatment alone [33]. The median time to remission was three months for JAK inhibitors, cyclosporine, and steroids, shorter than the six months observed with dupilumab [33]. However, a Cochrane review pointed out limitations in the current data, including the absence of direct comparisons between systemic treatments, limited data in paediatric populations, and uncertainties regarding long-term safety and efficacy compared to older systemic therapies or topical treatments [34].

Safety Considerations, Including Adverse Effects and Long-Term Use

Recent research has provided valuable insights into the treatment landscape for AD, focusing on various therapeutic approaches and their implications. Studies have highlighted the complexities surrounding using TCS versus TCIs in managing the condition. Evidence from randomized controlled trials involving over 2,570 children over a span of three to five years suggests that intermittent use of TCS may potentially increase the risk of non-skin infections, compromise vaccine responses, and even contribute to the development of lymphoma and other malignancies when compared to TCIs [35]. Conversely, a separate long-term trial spanning five years with 1,213 patients using mild to moderate potency TCS reported minimal risk of skin atrophy, supporting the safety of intermittent TCS use for managing acute flares of AD [35].

Dupilumab, an anti-IL-4Rα monoclonal antibody, has become a pivotal therapy for managing moderate-to-severe AD. Findings from a comprehensive five-year open-label extension trial involving 2,677 adults underscored dupilumab's favourable long-term safety profile, consistent with earlier placebo-controlled studies [36]. The study revealed that exposure-adjusted rates of treatment-emergent adverse events did not escalate over time and were lower than those observed in initial analyses of both the extension trial and a preceding 52-week placebo-controlled trial [36].

JAK inhibitors, such as ruxolitinib, have garnered attention for their potential benefits in AD treatment, but concerns regarding their safety profile have also emerged. Studies have indicated that JAK inhibitors may influence the progression of cutaneous lymphomas, necessitating careful monitoring and further investigation into their long-term effects [37]. A systematic review and meta-analysis have further highlighted potential adverse effects associated with JAK inhibitors, including elevated levels of creatine phosphokinase in patients with moderate-to-severe AD [37]. These findings underscore the importance of ongoing research and vigilance in understanding the full spectrum of risks and benefits associated with JAK inhibitors in clinical practice.

In emerging biologics, ongoing clinical trials, such as those evaluating astegolimab, hold promise for expanding treatment options for moderate-to-severe AD. A recent phase II trial involving 65 patients has completed its evaluation. However, specific outcomes have yet to be published, indicating ongoing efforts to explore novel therapies that could offer new avenues for managing this challenging skin condition [30]. These developments underscore a dynamic landscape of research and innovation aimed at improving outcomes and quality of life for individuals affected by AD.

Future directions and challenges

Potential for Combination Therapies

Combining systemic therapies, such as biologics and JAK inhibitors, has shown potential to enhance treatment efficacy, particularly in cases where primary or secondary treatments have failed. The strategic pairing of long half-life drugs, such as biologics, with short half-life drugs like JAK inhibitors, represents a thoughtful approach to managing AD [38,39]. A case report detailed a patient with severe, treatment-resistant AD who achieved significant improvement after six months of combination therapy with tralokinumab (a biologic) and upadacitinib (a JAK inhibitor), following unsuccessful attempts with several previous monotherapies [39]. Combining therapies that target distinct inflammatory pathways may offer advantages over single treatments, reflecting the complex pathophysiology of AD and the potential benefits of combination approaches [39]. However, the current data on combining systemic therapies in AD remains limited. Further double-blind, placebo-controlled studies are essential to fully assess these combination strategies' clinical efficacy [40]. Close monitoring is crucial due to potential additive toxicities when combining therapies, necessitating careful management to mitigate risks [38]. It is also vital to avoid redundant targeting of the same therapeutic pathway to maximize treatment efficacy [38]. While initial findings suggest promise in managing severe, treatment-resistant cases of AD, larger controlled studies are needed to establish both the effectiveness and safety of combination therapies [40]. A strategic approach addressing diverse inflammatory pathways may be most beneficial in optimizing treatment outcomes for patients with this challenging condition.

Addressing Unmet Needs in Severe AD Management

Advancements in managing AD are contingent upon addressing several critical areas of concern. One fundamental aspect is the development of improved diagnostic criteria that can be widely implemented in clinical settings to enhance diagnostic accuracy [41]. Current diagnostic practices heavily rely on clinical experience, necessitating validated criteria that can reliably differentiate AD from other skin conditions. Establishing such criteria would enable healthcare providers to diagnose AD more consistently and initiate appropriate treatment promptly, thereby improving patient outcomes. The quest for biomarkers to diagnose AD and predict disease prognosis represents another crucial frontier. There is a notable absence of serologic markers that could serve as reliable indicators for diagnosing AD or assessing disease severity and progression [41]. Identifying and validating biomarkers would represent a significant leap forward, potentially revolutionizing diagnostic approaches and enabling more precise prognostic assessments. This, in turn, could facilitate the development of personalized treatment strategies tailored to individual patients' specific phenotypes and endotypes.

Personalized medicine approaches are increasingly recognized as pivotal in addressing the heterogeneity of AD. By targeting specific patient profiles based on phenotypic and endotypic characteristics, personalized treatments hold promise for achieving more effective long-term disease control [41]. This approach acknowledges the variability in how AD manifests and responds to treatments among individuals, emphasizing the need for tailored therapeutic interventions to optimize outcomes. While targeted biologics like dupilumab have heralded significant advances in AD treatment, there remains a pressing need for therapies that offer improved efficacy and safety profiles [42]. The approval of such biologics has marked a transformative shift in AD management, yet ongoing research aims to refine treatment options further to better meet patient needs. Combination therapy strategies, such as integrating TCS with systemic biologics, have demonstrated enhanced efficacy compared to monotherapy, underscoring the potential benefits of multifaceted treatment approaches in AD management [42]. Addressing the complex web of comorbidities associated with severe AD, such as allergies and mental health issues, is also critical for improving overall patient outcomes [41]. A holistic approach that considers and manages these interconnected health concerns alongside AD treatment can lead to more comprehensive care and a better quality of life for patients. Additionally, ensuring the affordability and accessibility of novel therapies, particularly for underserved populations, remains a persistent challenge that requires ongoing attention and advocacy [42]. Efforts to address these multifaceted aspects of AD management promise to advance the field and enhance care for individuals living with this chronic and often debilitating condition.

## Conclusions

In conclusion, the evolving landscape of pharmacological therapies for AD reflects significant strides towards more targeted and effective treatment options. From traditional approaches like TCS to innovative biologic agents and small molecule inhibitors, advancements have expanded therapeutic possibilities, particularly for patients with moderate to severe AD who are unresponsive to conventional treatments. Integrating biologic therapies targeting interleukin pathways, JAK inhibitors, and PDE-4 inhibitors represents promising avenues for managing AD by addressing underlying immunological dysregulation and skin barrier dysfunction. Furthermore, the potential for personalized medicine approaches, leveraging biomarkers and genotype-based strategies, holds promise for tailoring treatment regimens to individual patient profiles, optimizing outcomes, and minimizing adverse effects. Despite these advancements, challenges such as long-term safety profiles, accessibility, and cost-effectiveness remain pertinent. Future research should continue to explore combination therapies, refine personalized treatment algorithms, and address these challenges to further enhance AD management and improve the quality of life for affected individuals.

## References

[1] Kolb L, Ferrer-Bruker SJ (2024). Atopic dermatitis. StatPearls [Internet].

[2] Kelly KA, Balogh EA, Kaplan SG, Feldman SR (2021). Skin disease in children: effects on quality of life, stigmatization, bullying, and suicide risk in pediatric acne, atopic dermatitis, and psoriasis patients. Children (Basel).

[3] Tripathi PN, Lodhi A, Rai SN (2024). Review of pharmacotherapeutic targets in Alzheimer’s disease and its management using traditional medicinal plants. Degener Neurol Neuromuscul Dis.

[4] Hajjo R, Sabbah DA, Abusara OH, Al Bawab AQ (2022). A review of the recent advances in Alzheimer’s disease research and the utilization of network biology approaches for prioritizing diagnostics and therapeutics. Diagnostics (Basel).

[5] Gelbard CM, Hebert AA (2008). New and emerging trends in the treatment of atopic dermatitis. Patient Prefer Adherence.

[6] Chew YL, Al-Nema M, Ong VWM (2018). Management and treatment of atopic dermatitis with modern therapies, complementary and alternative medicines: a review. Orient Pharm Exp Med.

[7] Choopani R, Mehrbani M, Fekri A, Mehrabani M (2017). Treatment of atopic dermatitis from the perspective of traditional Persian medicine: presentation of a novel therapeutic approach. J Evid Based Complementary Altern Med.

[8] (2024). Holistic treatment techniques for managing atopic dermatitis. https://www.drugtopics.com/view/holistic-treatment-techniques-for-managing-atopic-dermatitis.

[9] Alexander H, Patton T, Jabbar-Lopez ZK, Manca A, Flohr C (2019). Novel systemic therapies in atopic dermatitis: what do we need to fulfil the promise of a treatment revolution?. F1000Res.

[10] (2024). Current landscape of atopic dermatitis (AD). https://www.dermatologytimes.com/view/current-landscape-of-atopic-dermatitis-ad-.

[11] (2024). Treatment landscape surrounding atopic dermatitis. https://www.ajmc.com/view/treatment-landscape-surrounding-atopic-dermatitis.

[12] Boytsov NN, Gorritz M, Wang X, Malatestinic WN, Wade RL, Goldblum OM (2022). The current treatment landscape in adult atopic dermatitis in the United States: results from a cross-sectional real-world study. J Dermatolog Treat.

[13] Souto EB, Dias-Ferreira J, Oliveira J (2019). Trends in atopic dermatitis—from standard pharmacotherapy to novel drug delivery systems. Int J Mol Sci.

[14] Goddard AL, Lio PA (2015). Alternative, complementary, and forgotten remedies for atopic dermatitis. Evid Based Complement Alternat Med.

[15] Wu AY, Sur S, Grant JA, Tripple JW (2019). Interleukin-4/interleukin-13 versus interleukin-5: a comparison of molecular targets in biologic therapy for the treatment of severe asthma. Curr Opin Allergy Clin Immunol.

[16] Massey O, Suphioglu C (2021). Recent advances in the inhibition of the IL-4 cytokine pathway for the treatment of allergen-induced asthma. Int J Mol Sci.

[17] Słuczanowska-Głąbowska S, Ziegler-Krawczyk A, Szumilas K, Pawlik A (2021). Role of Janus Kinase inhibitors in therapy of psoriasis. J Clin Med.

[18] Tampa M, Mitran CI, Mitran MI, Georgescu SR (2023). A new horizon for atopic dermatitis treatments: JAK inhibitors. J Pers Med.

[19] (2024). Janus kinase inhibitors. https://dermnetnz.org/topics/janus-kinase-inhibitors.

[20] Li H, Zuo J, Tang W (2018). Phosphodiesterase-4 inhibitors for the treatment of inflammatory diseases. Front Pharmacol.

[21] Crocetti L, Floresta G, Cilibrizzi A, Giovannoni MP (2022). An overview of PDE4 inhibitors in clinical trials: 2010 to early 2022. Molecules.

[22] Ivey AG (2024). Phosphodiesterase-4 inhibitors for psoriasis. https://www.webmd.com/skin-problems-and-treatments/psoriasis/pde-4-inhibitors-psoriasis.

[23] Crowley EL, Gooderham MJ (2023). Phosphodiesterase-4 inhibition in the management of psoriasis. Pharmaceutics.

[24] Hernandez TD, Aleman SJ, Bao-Loc-Trung M (2024). Advancing treatment in atopic dermatitis: a comprehensive review of clinical efficacy, safety, and comparative insights into corticosteroids, calcineurin inhibitors, and phosphodiesterase-4 inhibitors as topical therapies. Cureus.

[25] Qi HJ, Li LF (2021). New biologics for the treatment of atopic dermatitis: analysis of efficacy, safety, and paradoxical atopic dermatitis acceleration. Biomed Res Int.

[26] Nankervis H, Thomas KS, Delamere FM, Barbarot S, Rogers NK, Williams HC (2016). Topical corticosteroids and topical immunomodulators. Scoping Systematic Review of Treatments for Eczema.

[27] Solimani F, Meier K, Ghoreschi K (2019). Emerging topical and systemic JAK inhibitors in dermatology. Front Immunol.

[28] Silverberg JI, Armstrong A, Blauvelt A, Reich K (2023). Assessment of efficacy and safety outcomes beyond week 16 in clinical trials of systemic agents used for the treatment of moderate to severe atopic dermatitis in combination with topical corticosteroids. Am J Clin Dermatol.

[29] Park CO, Kim SM, Lee KH, Bieber T (2024). Biomarkers for phenotype-endotype relationship in atopic dermatitis: a critical review. eBioMedicine.

[30] (2024). A study to look at the effects of a new medicine (astegolimab) in comparison to placebo - in patients with eczema (atopic dermatitis). https://forpatients.roche.com/en/trials/autoimmune-disorder/a-study-to-assess-the-efficacy-and-safety-of-mstt1041a--54098.html.

[31] Müller S, Maintz L, Bieber T (2024). Treatment of atopic dermatitis: recently approved drugs and advanced clinical development programs. Allergy.

[32] Drucker AM, Morra DE, Prieto-Merino D (2022). Systemic immunomodulatory treatments for atopic dermatitis: update of a living systematic review and network meta-analysis. JAMA Dermatol.

[33] Sato E, Arima H, Ito K, Iwata M, Imafuku S (2024). Comparative effectiveness of treatments on time to remission in atopic dermatitis: real-world insights. J Cutan Immunol Allergy.

[34] Banerjee N, Grewal A (2021). What is the most effective systemic treatment for atopic dermatitis?. Clin Exp Allergy.

[35] Harvey J, Lax SJ, Lowe A (2023). The long-term safety of topical corticosteroids in atopic dermatitis: a systematic review. Skin Health Dis.

[36] (2024). Long-term safety of dupilumab in adults with moderate to severe atopic dermatitis. https://www.dermatologytimes.com/view/long-term-safety-of-dupilumab-in-adults-with-moderate-to-severe-atopic-dermatitis.

[37] Kołkowski K, Trzeciak M, Sokołowska-Wojdyło M (2021). Safety and danger considerations of novel treatments for atopic dermatitis in context of primary cutaneous lymphomas. Int J Mol Sci.

[38] (2024). Combining systemic therapies in psoriasis and atopic dermatitis. https://www.dermatologytimes.com/view/combining-systemic-therapies-in-psoriasis-and-atopic-dermatitis.

[39] Lansang RP, Zhao IX, Lansang P (2023). Atopic dermatitis treated with tralokinumab and upadacitinib combination therapy: a case report. SAGE Open Med Case Rep.

[40] Nahm DH, Lee ES, Park HJ, Kim HA, Choi GS, Jeon SY (2008). Treatment of atopic dermatitis with a combination of allergen-specific immunotherapy and a histamine-immunoglobulin complex. Int Arch Allergy Immunol.

[41] Lobefaro F, Gualdi G, Di Nuzzo S, Amerio P (2022). Atopic dermatitis: clinical aspects and unmet needs. Biomedicines.

[42] (2024). Unmet needs in the treatment of atopic dermatitis. https://www.dermatologytimes.com/view/unmet-needs-in-the-treatment-of-atopic-dermatitis.

